# Nano-Size Biomass Derived from Pomegranate Peel for Enhanced Removal of Cefixime Antibiotic from Aqueous Media: Kinetic, Equilibrium and Thermodynamic Study

**DOI:** 10.3390/ijerph17124223

**Published:** 2020-06-13

**Authors:** Mehdi Esmaeili Bidhendi, Zahra Poursorkh, Hassan Sereshti, Hamid Rashidi Nodeh, Shahabaldin Rezania, Muhammad Afzal Kamboh

**Affiliations:** 1 School of Environment, College of Engineering, University of Tehran, Tehran 14155-6135, Iran; esmaeilib@ut.ac.ir; 2 Department Chemistry, Faculty of Science, University of Tehran, Tehran 14155-6135, Iran; Sara.poursorkh@gmail.com; 3 Food Technology and Agricultural Products Research Centre, Standard Research Institute (SRI), Karaj 31745-139, Iran; rnhamid2@gmail.com; 4 Department of Environment and Energy, Sejong University, Seoul 05006, Korea; 5 Department of Chemistry, Shaheed Benazir Bhutto University, Shaheed Benazirabad, Sindh 67450, Pakistan; chairman_chemistry@sbbusba.edu.pk

**Keywords:** pomegranate peel activated carbon, cefixime micro-pollutant, kinetic, equilibrium, thermodynamic

## Abstract

Nano-sized activated carbon was prepared from pomegranate peel (PG-AC) via NaOH chemical activation and was fully characterized using BET, FT-IR, FE-SEM, EDX, and XRD. The newly synthesized PG-AC was used for cefixime removal from the aqueous phase. The effective parameters on the adsorption process, including solution pH (2–11), salt effect (0–10%), adsorbent dosage (5–50 mg), contact time (5–300 min), and temperature (25–55 °C) were examined. The experimental adsorption equilibrium was in close agreement with the type IV isotherm model set by the International Union of Pure and Applied Chemistry (IUPAC). The adsorption process was evaluated with isotherm, kinetic, and thermodynamic models and it is were well fitted to the Freundlich isotherm (*R*^2^ = 0.992) and pseudo-second-order model (*R*^2^ = 0.999). The Langmuir isotherm provided a maximum adsorption capacity of 181.81 mg g^−1^ for cefixime uptake onto PG-AC after 60 min at pH 4. Hence, the isotherm, kinetic and thermodynamic models were indicated for the multilayer sorption followed by the exothermic physical adsorption mechanism.

## 1. Introduction

Antibiotics as emerging pollutants are a major significant global concern since their widespread use as prescription drugs and their potently bioactive properties render them a direct threat to public health from uncontrolled exposures via contaminated drinking water [1,2,3]. Antibiotics are a large group of medicines including penicillin, cephalosporins (cefixime), fluoroquinolones, aminoglycosides, monobactams, carbapenems, and tetracyclines [4,5]. These antibiotics are an important part of modern life because of their vital role in the treatment of a wide range of human and animal infections [6,7]. One common antibiotic used widely in the world is cefixime, which is useful for combating a number of serious bacterial infections such as pneumonia and urinary tract diseases [8,9]. Unfortunately, despite the clinical benefits of antibiotics, some of them are not completely metabolized by humans or animals during consumption, resulting in wide side effects [10,11]. Therefore, discharging antibiotics into the aqueous environments (rivers, groundwater, and lakes) without adequate treatment results in irreparable harm to human health, especially the promotion of antibiotic resistance of pathogenic microorganisms [12,13], among populations who drink it on a regular basis.

Based on the frequent inability of conventional wastewater treatment methods to remove or reduce the number of unwanted antibiotics to the published control standards, several more effective treatment methods based on photocatalytic degradation [14,15,16], oxidation, biodegradation, electro-degradation, and adsorption have been introduced [17,18]. Adsorption is considered to be the most attractive treatment method owing to its significant benefits such as economic features, reliability, and its ability to prevent the entry of secondary toxic substances by restricting their transfer into the aquatic environment [19,20]. Researchers have studied the capabilities of different adsorbents in relation to antibiotic adsorption such as metal-organic framework, carbon-based material, montmorillonite, zeolite, graphene oxide and activated carbon [21,22,23,24]. Owing to low-cost, a high density of active sites, significant adsorption capacity, and a high specific surface area, activated carbons (ACs) are proper adsorbent for pollutant removal of particulate antibiotics [22,25]. Despite the applicability of commercial ACs for wastewater treatment and contaminant removal from aqueous environments, commercial ACs are an expensive option [26,27]. Consequently, research in recent years has sought to produce ACs from affordable natural biomass materials from agricultural wastes such as coconut shell, papaya peel, vine wood, macadamia nut shells, coffee endocarp, olive stones, and walnut shells [28,29,30]. Regeneration of adsorbents is the key parameter: which ACs can be regenerated with low cost thermal and hydrothermal processes after saturated with pollutants [31,32]. Based on literature, pomegranate based biomass is widely used in pollutant remediation owing to cost-effectiveness [33], eco-friendliness [34], remarkable specific surface area [35], biosorption of malachite green [36], and a cationic and anionic nature [37].

NaOH has been widely used as promising chemical reagent for preparation of AC with high porosity and uniform shape, pore size distribution, and mesopore volumes [38,39]. In this study, pomegranate peel was used as an AC source, activated via NaOH, for the removal of cefixime in aqueous media. The effective parameters of the removal process were optimized to achieve the best possible performance. In addition, the adsorption kinetics of the removal was investigated with pseudo-first-order, pseudo-second-order, and intra-particle diffusion (Weber–Morris model) models to obtain the most suitable model to understand the adsorption mechanism.

## 2. Experimental

### 2.1. Reagents and Materials

All chemicals used were of analytical grade. Sodium hydroxide (NaOH), hydrochloric acid (HCl, 37%) and sodium chloride (NaCl) were purchased from Merck Chemicals (Darmstadt, Germany).

### 2.2. Instruments

To evaluate the surface morphology of PG-AC, a MIRA3 TESCAN (Prague, Czech Republic) and a Carl Zeiss Supra 35-VP (Oberkochen, Germany) field emission-scanning electron microscope (FE-SEM) were used. A Bruker Equinox 55 FT-IR spectrometer (Bremen, Germany) was operated in the wavelength range of 450 to 4000 cm^−1^ for the investigation of functional groups of PG-AC. A Bruker X-ray diffractometer (Bremen, Germany) was used for crystallinity studies of PG-AC in the 2 theta range of 10° to 90° with CuK radiation (λ = 1.5418 Å). Brunauer-Emmett-Teller (BET) specific surface area, pore volume, and pore size of the prepared PG-AC were investigated using a Belsorp-mini II, BEL Japan Inc. (Osaka, Japan) under N_2_ gas (28.01 g mol^−1^).

### 2.3. Preparation of Nano-Sized Activated Carbon

Activation of carbon was conducted by following the procedure described previously [40]. The raw pomegranate peel (PG) was washed, dried, and powdered. Then, 50 g of the powder was placed in the furnace and heated at 400 °C for 2 h. The carbonized material was mixed with different ratios of NaOH (w:w%) in 20 mL of distilled water, stirred for 2 h, and dried at 130 °C for 4 h. Then, the dried product was heated in the furnace at 810 °C for 2 h. The activated carbon (PG-AC) was cooled and washed with distilled water. Different ratios of NaOH:PG-AC (1:1, 1:3, 1:5, and 1:7) were labeled as PG-AC1, PG-AC3, PG-AC5, and PG-AC7 for further experiments.

### 2.4. Adsorption Process

The adsorption efficiency of the prepared activated carbon for removal of cefixime was evaluated in batch experiments. The effects of pH (2–11), contact time (10–300 min), ionic strength (0–10%), and adsorption isotherms (10–200 mg L^−1^) on the efficiency of the method were studied carefully. The adsorption behavior (isothermal) of the adsorbent was analyzed by adding 50 mg samples of the adsorbent to 20 mL samples of cefixime solution at different concentrations. After 60 min (equilibrium conditions), the adsorbent was removed from the sample solution. The residual concentration of cefixime in the aqueous phase was measured using UV-vis spectrophotometry. Cefixime concentration adsorbed per unit mass of adsorbent (*q_e_*) and the removal efficiency (*R%*) was calculated by Equations (1) and (2), respectively.

(1) $$R\%=\frac{C_{0}-C_{e}}{C_{e}}\times 100$$

(2) $$q_{e}=\frac{C_{0}-C_{e}}{m}\times V$$

where *q_e_* (mg g^−1^), *C_0_,* and *C_e_* are the equilibrium adsorption capacity and the concentration of cefixime in the aqueous phase before and after adsorption, respectively (mg L^−1^), *V* is the volume of the aqueous phase (mL) and *m* is the mass of the used adsorbent (g).

In order to investigate the regeneration of PG-AC, 50 mg of the activated carbon (cefixime 20 mg L^−1^) was placed in a test tube with 3 mL methanol and the admixture was shaken for 30 min. The concentration of desorbed cefixime was measured with UV-vis spectrophotometry. The activated carbon was washed with distilled water for reuse in further experiments.

## 3. Results and Discussion

### 3.1. Characterization

#### 3.1.1. BET Analysis

**Figure 1:**
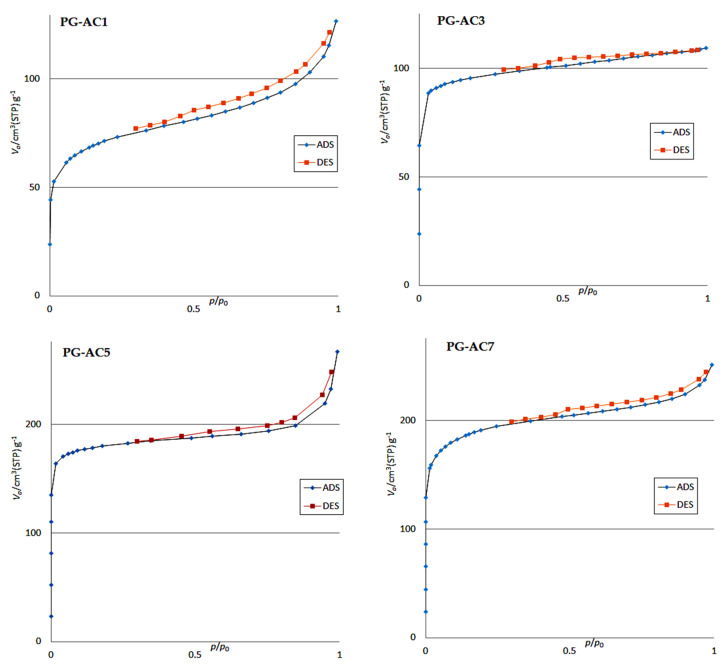
Adsorption-desorption isotherm for BET analysis with different ratios of NaOH:PG-AC (PG-AC1, 1:1), (PG-AC3, 1:3), (PG-AC5, 1:5) and (PG-AC7, 1:7).

The specific surface area of the prepared plain carbon and the activated carbon samples (PG-AC1, PG-AC3, PG-AC5, and PG-AC7) with different ratios of NaOH:PG-AC (1:1, 1:3, 1:5 and 1:7) were analyzed using the Brunauer-Emmett-Teller (BET) technique (Figure 1). The results showed that the BET surface area values obtained for the prepared plain carbon (9.69 m^2^ g^−1^) and activated PG-AC1, PG-AC3, PG-AC5, and PG-AC7 samples were 159, 231, 694, and 455 m^2^ g^−1^, respectively. The obtained pore diameter distribution values were 13.41 nm, 4.23 nm, 3.01 nm, 2.33 nm, and 3.37 nm for the plain carbon, PG-AC1, PG-AC3, PG-AC5, and PG-AC7, respectively. Pore distributions indicated that the prepared materials were mesoporous in nature (2 < pore diameter < 50 nm), PG-AC5 had the highest specific surface area, and the mesoporous structure was selected for further adsorption experiments and characterization.

#### 3.1.2. FT-IR Spectroscopy

**Figure 2:**
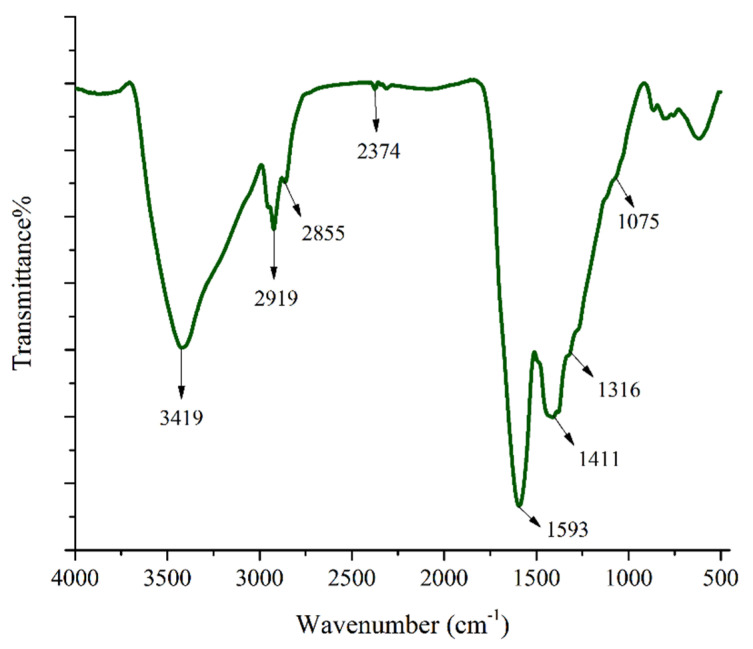
The FT-IR spectrum of PG-AC5 nanomaterial.

Surface functional groups of the prepared activated carbon were studied with IR spectroscopy. Figure 2 shows the FT-IR spectrum of PG-AC5 and the absorption band at 3419 cm^−1^ is associated with OH stretching vibration of hydroxyl functional groups, the peaks at 2929 and 2855 cm^−1^ are related to C-H stretching vibrations, and the strong band at 1593 cm^−1^ corresponds to stretching of the aromatic rings (aromatic C-C and C=C vibrations). Also, the double band at 1411 and 1316 cm^−1^ can be attributed to oxygen-containing functional groups, possibly O-H of alcohols [41] or in-plane vibration of the O-H bond in carboxylic groups [42]. The band at 1075 cm^−1^ corresponds to C-O stretching of primary alcohol groups. These results of FT-IR and EDX proved the expected activated carbon was prepared successfully.

#### 3.1.3. X-ray Diffractometry

**Figure 3:**
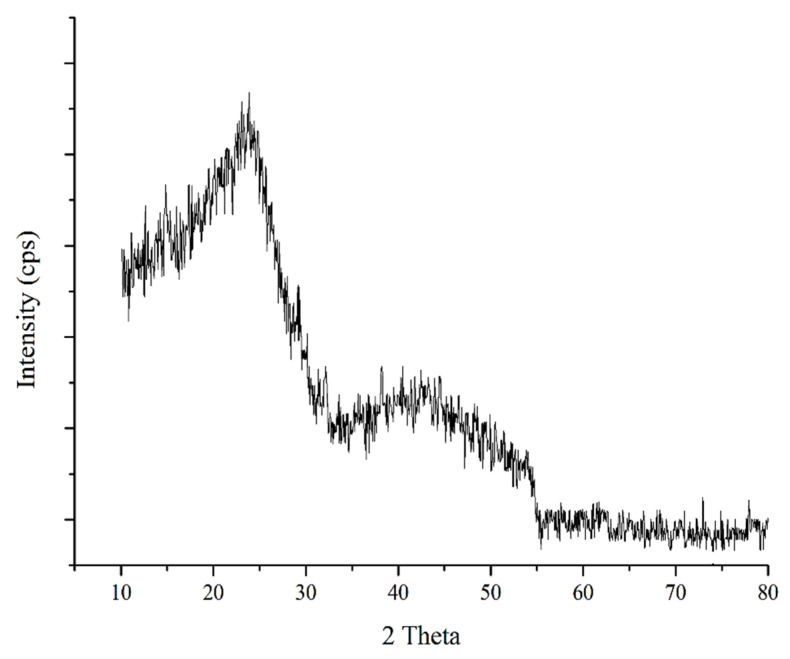
The XRD pattern of pomegranate peel activated carbon.

The crystalline structure of PG-AC5 was investigated using the XRD technique. As shown in Figure 3, there was no specific pattern in the spectrum, therefore the prepared powder has an amorphous structure.

#### 3.1.4. Field Emission Scanning Electron Microscopy

**Figure 4:**
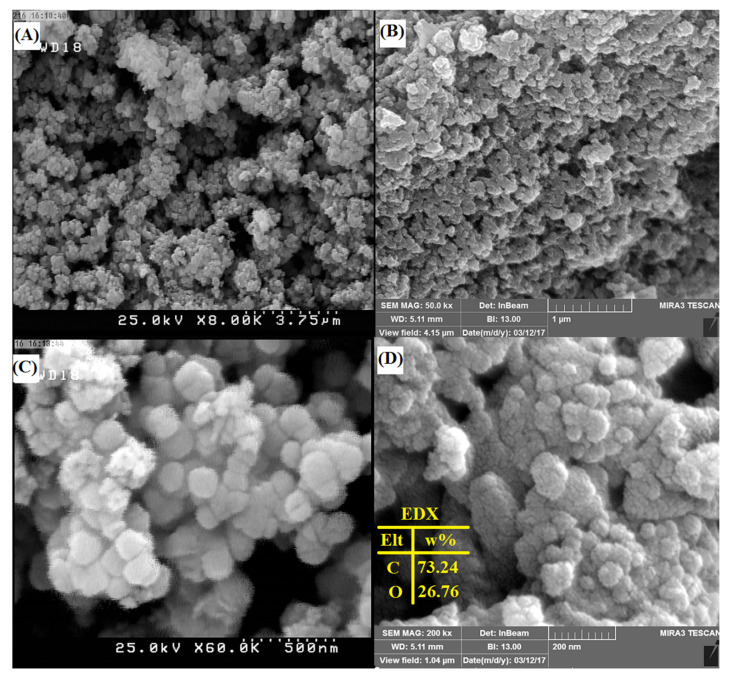
The FE-SEM micrographs of PG-AC5 with different scales (**A**) 3.75 µm, (**B**) 1 µm, (**C**) 500 nm and (**D**) 200 nm.

The surface morphological characteristics of PG-AC were studied by FE-SEM. Figure 4A,B show an irregular external surface full of cavities for the activated carbon. In addition, the particles are almost spherical with an average diameter of around 150 nm. Furthermore, the images indicate a porous structure with a high surface area, thus providing a high adsorption capacity. Figure 4C,D show with higher magnification that the surface of the newly synthesized material is uniform and there were no aggregations or beads on its surface. The results of the EDX analysis are also shown in Figure 4D. The EDX results indicated that the two major elements forming on PG-AC were carbon (73.24%) and oxygen (26.76%), and that the proposed biomass was prepared successfully.

### 3.2. Effect of pH

**Figure 5:**
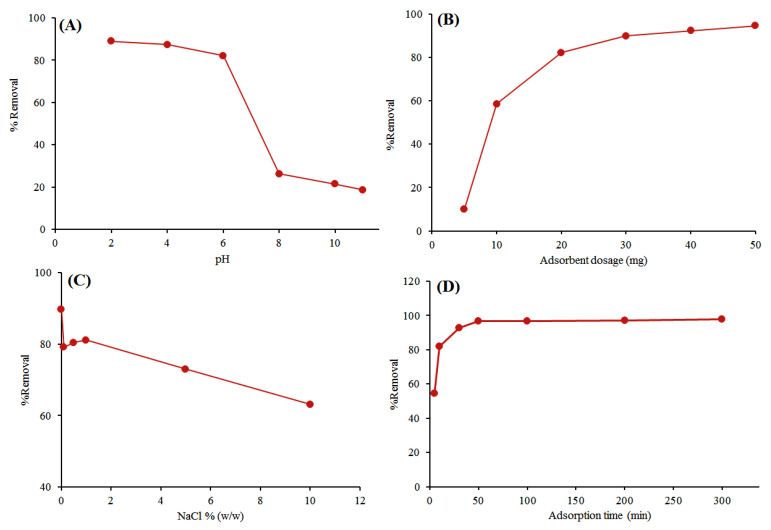
Effect of (**A**) solution pH, (**B**) adsorbent dosage, (**C**) NaCl percentage and (**D**) contact time on cefixime removal efficiency.

The pH of the sample solution may affect the solubility of cefixime, its chemical structure, the sorbent surface charge, and thus the adsorption efficiency [43]. Therefore, the effect of pH in the range of 2–11 on the percentage of cefixime removal was studied. Figure 5A shows that high removal efficiency for cefixime is at pH 2–6, then declines. Removal efficiency was decreased dramatically by increasing the pH to more than 6. In addition, with regard to pK_a_ of cefixime (pK_a_ 2.5) [44] and Le Chatelier’s principle, cefixime itself is a weak acid and may be found in the neutral form in acidic pHs. All these factors proved that the maximum adsorption of cefixime occurs in acidic conditions because of hydrogen bonding and possible electrostatic interactions between activated carbon and this antibiotic. Therefore, pH 4 was selected for further experiments.

### 3.3. Effect of Adsorbent Dosage

The adsorbent dosage can directly affect the efficiency of the method and the adsorption capacity [43]. The influence of the adsorbent dose on removal efficiency was explored by testing various adsorbent dosages in the range of 5–50 mg. The experiments were performed using standard cefixime solution of 50 mg L^−1^ at room temperature, a contact time of 1 h, and pH 3. Figure 5B shows that by increasing the adsorbent dosage from 5 to 50 mg the removal percentage enhanced from 49 to 95%. By occupying the available active sites of the adsorbent, the removal efficiency remained virtually constant for adsorbent dosages greater than 50 mg.

### 3.4. Effect of Salt Concentration

The influence of the ionic strength on the removal efficiency was studied from 0 to 10% at the optimized conditions (adsorption time of 1 h, pH of 3, 50 mg adsorbent and cefixime concentration of 50 mg L^−1^). The results in Figure 5C show that adding up to 1% NaCl had no significant influence on the adsorption of cefixime on AC. It was observed that adsorption decreased to 62% by the further addition of NaCl up to 10%. The latter effect probably decreases the strong electrostatic interactions between cefixime and AC, and this is probably due to competition of Cl anions or Na cations with the functional groups of cefixime to occupy the active sites. Therefore, for further experiments the effect of salt (NaCl) was ignored.

### 3.5. Adsorption Time

The contact time between the adsorbent and the sample solution is a key factor for the enhanced removal of the target analyte. The influence of this factor on the removal of cefixime was studied in the range of 10–300 min. The concentration of cefixime, pH, and adsorbent dosages was adjusted to 50 mg L^−1^, 3 and 50 mg, respectively. Figure 5D shows that during the first 50 min, the percentage of cefixime removal increased rapidly and then the system reached equilibrium. This is probably due to the presence of large available active sites at the beginning of the adsorption process.

### 3.6. Adsorption Kinetics

**Figure 6:**
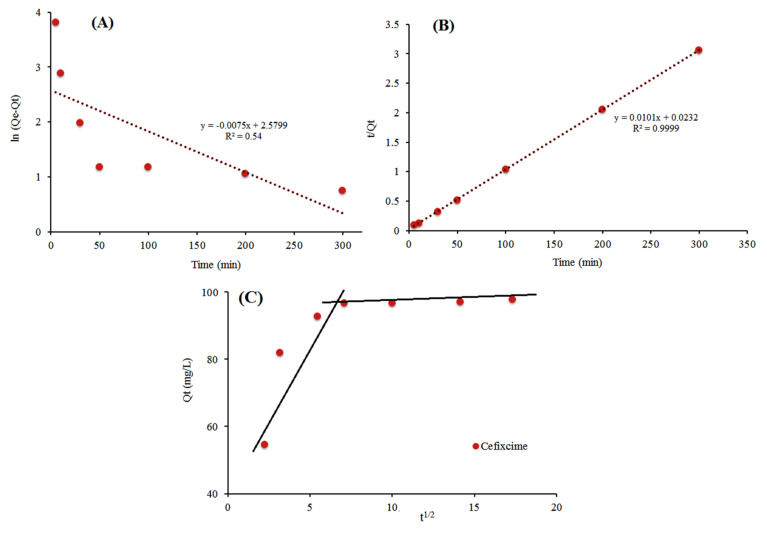
Adsorption kinetic models for (**A**) pseudo-first-order, (**B**) pseudo-second-order, and (**C**) intra-particle diffusion.

The experimental data for contact time (Figure 5D) was evaluated with kinetic models, which is useful to describe the adsorption mechanism and mass transfer rate on the surface or interior sites. The adsorption kinetics of cefixime onto the activated carbon was investigated with three well known models of pseudo-first-order, pseudo-second-order and intra-particle diffusion (Weber–Morris model) models. The linear form of the proposed models is described by Equations (3)–(5).

(3) $$\ln\left(q_{e}-q_{t}\right)=lnq_{e}-k_{1}t$$

(4) $$\frac{t}{q_{t}}=\frac{1}{k_{1}q_{e}^{2}}+\frac{t}{q_{e}}$$

(5) $$q_{t}=k_{id}t^{1/2}+C_{i}$$

where *q_e_* (mg g^−1^) is the equilibrium adsorption capacity, *q_t_* (mg g^−1^) is the adsorption capacity at different times, and *C_i_* is the thickness of the boundary layer of intra-particle diffusion. The *k*_1_ (min^−1^), *k*_2_ (g mg^−1^ min^−1^), and *k_id_* are model constants. The values of kinetic parameters can be obtained from the slope and intercept of the linearized form of proposed models as shown in Figure 6A–C.

Table 1 illustrates the kinetic models and their parameters, which are obtained from linear plots in Figure 6A,B. Regarding the *R*^2^ values, the pseudo-second-order model (R^2^ = 0.9999) best fit the experimental data as compared pseudo-first-order (R^2^ = 0.5418). In addition, the theoretical *q_e_* (calculated) based on pseudo-second-order is closer to the *q_e_* (experimental) compared to the pseudo-first-order. Thus, a pseudo-second-order model is applicable to evaluation of the cefixime kinetic onto the activated carbon. Previous studies suggested that the pseudo-second-order model probably follows a chemical sorption mechanism for adsorption through electron sharing between analytes and adsorbent [45]. Figure 6C presents a multilinearity sorption process for cefixime: the first sharp step is surface adsorption due to the fast mass transfer from solution to the adsorbent surface [46] and the second step is attributed to the equilibrium sorption on the interior sites of the adsorbent. In the second step, the high values of the *C_i2_* also suggest the abundance of adsorbate (analyte) in the boundary layer [47]. This suggests that the cefixime uptake onto PG-AC takes place faster on the surface than on internal sites.

### 3.7. Initial Concentration and Adsorption Isotherm

**Figure 7:**
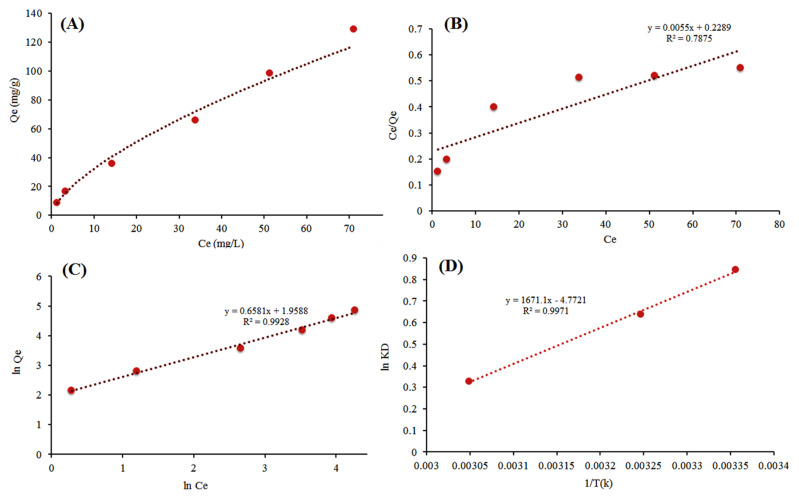
(**A**) Experimental adsorption isotherm for cefixime adsorption onto PG-AC5, (**B**) Langmuir isotherm, (**C**) Freundlich isotherm and (**D**) thermodynamic model.

The equilibrium isotherm was investigated by varying the antibiotic concentration from 10–200 mg L^−1^. The batch adsorption process was conducted using 50 mg PG-AC5 at pH 3 for 60 min shaking time at room temperature (Figure 7A). The experimental isotherm equilibrium (A) confirms that the adsorption capacity increased from 5 to 120 mg g^−1^ by increasing the initial concentration, since a larger amount of analytes was available to load onto the adsorbent active sites. Hence, the adsorption of cefixime onto the PG-AC5 (Figure 7A) was found to be in agreement with Type IV and V adsorption models set by IUPAC [48]. These types of models are relevant to the multilayer adsorption process.

An equilibrium isotherm was performed to evaluate the Langmuir and Freundlich models, seeking to explain the adsorption capacity, the heat of sorption, adsorbent surface heterogeneity, and sorption pattern. The linear form of Langmuir and Freundlich isotherm is expressed under Equations (6) and (7) as follows:

(6) $$\frac{C_{e}}{q_{e}}=\frac{C_{e}}{q_{m}}+\frac{1}{k_{1q_{m}}}$$

(7) $$lnq_{e}=\ln K_{F}+\left(1/n\right)\ln C_{e}$$

where *C_e_* (mg L^−1^) is the residual concentration of cefixime in solution, *q_m_* (mg g^−1^) is maximum adsorption capacity, *k_L_* is Langmuir constant (describing the heat of adsorption), *K_F_* [(mg g^−1^) (L/mg)^1/n^] and 1/*n* are the Freundlich constants (intensity of adsorption and heterogeneity factor).

Table 2 and Figure 7B,C illustrate that the adsorption of cefixime onto PG-AC was found to fit well with the Freundlich isotherm, as the Freundlich isotherm provided a higher *R*^2^ compared to the Langmuir isotherm. Additionally, Freundlich (*n* = 1.52) describes the adsorption of cefixime that occurred on the heterogeneous surface. It suggests a multilayer adsorption pattern for cefixime adsorption onto PG-AC, which was in agreement with the IUPAC protocol.

### 3.8. Effect of Temperature and Thermodynamics

The effect of temperature on the cefixime adsorption process was investigated at three levels: 25 °C, 35 °C, and 55 °C at pH 3, using 50 mg of dosage, 60 min shaking time and concentration of 100 mg L^−1^ (Figure 7D). A thermodynamic model was performed to evaluate the enthalpy (ΔH), entropy (ΔS), and Gibbs free energy (ΔG) and explain the nature and mechanism of the adsorption of cefixime onto PG-AC. These parameters are described by Van’t Hoff equation in Equations (8) and (9).

(8) $$lnK_{D}=-\frac{\Delta H}{RT}+\frac{\Delta S}{R}$$

(9) $$-\Delta G=-RT\;ln\;K_{D}$$

where *T(K)*, *R* (0.0083145 kJ mol^−1^ K^−1^) and *K_D_* are temperatures, universal gas constant, and the thermodynamic distribution coefficient (*ln K_D_ = q_e_/C_e_*), respectively [49].

Table 3 reveals that removal efficiency did not change significantly at different temperatures. In contrast, adsorption capacity decreased from 69.97 to 58.13 mg g^−1^ when the temperature increased from 25 to 55 °C. The effect of increasing temperature decreased the feasibility of adsorption at high temperatures. The negative values of ∆G, ΔH and ∆S revealed the spontaneous sorption, exothermic nature, and randomness of adsorption for cefixime adsorption onto PG-AC, respectively. The obtained ΔG values are lower than -18 kJ mol^−1^, which suggests that the adsorption for cefixime was physical rather than chemical.

### 3.9. Regeneration and Recovery

The adsorption-desorption cycle was repeated fifteen times based on regeneration methodology. The results indicated that the removal efficiency does not decrease significantly up to ten cycles (removal > 87%). Thus, the adsorbent can be reused for ten times without a significant loss of removal efficiency for water containing 20 mg L^−1^ cefixime. This result is comparable with other studies in which activated-carbon-based biomass regenerated 4 times (100 mg L^−1^) [33], 5 times (500 mg L^−1^) [32], 4 times (200 mg L^−1^) [50] and 5 times (231 mg L^−1^) [51].

### 3.10. Comparison with Other Adsorbents

In order to evaluate the sorption efficiency of PG-AC toward cefixime, its ability was compared with other materials such as the adsorption of Portland cement, GO/MNPs–SrTiO_3_, vine wood AC, Biomass-AC, and MGO4 [52,53,54,55]. The comparison was conducted in terms of pH, adsorption time, and adsorption capacity of antibiotics on various materials as shown in Table 4. Comparative studies indicate that PG-AC provided a high adsorption capacity (181.81 mg g^−1^) compared to the other materials, while the lowest was Portland cement (<6 mg g^−1^) and the highest was biomass-AC (<166 mg g^−1^). By considering the adsorption time, PG-AC provided fast adsorption compared to Portland cement, vine wood AC and bentonite. Thus, prepared PG-AC is a suitable adsorbent for the efficient and effective adsorption of cefixime antibiotics from aqueous environments.

## 4. Conclusions

In this study, a new nano-sized PG-AC adsorbent was prepared and its performance for the adsorption of cefixime antibiotic from aqueous media was evaluated. Freundlich models and thermodynamic findings showed that the cefixime uptake follows a multilayer sorption pattern under a physical adsorption process with an endothermic nature. A pseudo-second-order kinetic model provided the best fit for experimental adsorption time and intra-particle diffusion was attributed to equilibrium sorption occurring on the interior sites of the adsorbent. Finally, PG-AC was found to be an efficient and effective adsorbent for the rapid (60 min) and effective adsorption of cefixime antibiotics with 95% removed from aqueous environments. Thus, this study proved that pomegranate-based biomass can be used as an alternative adsorbent for remediation of pharmaceutical residuals.

## Figures and Tables

**Table 1:** The pseudo-first-order, pseudo-second-order and intra-particle diffusion parameters for the adsorption of cefixime (experimental *q_e_* was 100.1 mg g^−1^).

Model	Parameters	Cefixime
Pseudo first order	*q_e_* (mg/g)	12.84
	*k*_1_ (1/min)	0.0075
	*R* ^2^	0.5418
Pseudo second order	*q_e_* (mg/g)	99.09
	*k*_2_ (g/(mg·min))	0.0001
	*R* ^2^	0.9999
Intra-particle diffusion	*K_id,_*_1_	0.7622
	*C_i_*_1_	63.405
	*R*_1_^2^	0.6837
	*K_id,_*_2_	0.0047
	*C_i_*_2_	96.356
	*R*_2_^2^	0.9153

**Table 2:** Adsorption isotherm models and parameters for the adsorption of cefixime.

Isotherms	Parameters	Cefixime
Langmuir	*q_m_* (mg/g)	181.81
	*k_L_* (L/mg)	0.024
	*R* ^2^	0.787
Freundlich	*K_F_* [(mg/g) (L/mg)^1/n^]	6.201
	*n*	1.52
	*R* ^2^	0.992

**Table 3:** Effect of temperature on removal efficiency and thermodynamic parameters.

Temp °C	Removal%	*q_e_* (mg/g)	∆G (kJ/mol)	ΔH (kJ/mol)	ΔS (kJ/mol K)
25	89	69.97	−2.09		
35	87	65.42	−1.63	−13.89	−0.038
55	85	58.13	−0.89		

**Table 4:** Different ACs used for the removal of antibiotics.

Adsorbent	Antibiotics	Time (min)	*q_e_* (mg/g)	Ref
Nano-activated carbon	Cefixime	60	181.81	This study
Portland cement	Cefixime	180	6.99	[52]
GO/MNPs–SrTiO_3_	Cefotaxime	30	18.21	[43]
Vine wood AC	Cephalexin	8 h	7.2	[53]
Biomass-AC	Norfloxacin	40	166.99	[54]
MGO	Tetracycline	20	39.1	[55]
